# Assessing Ischemic Injury in Human Intestine Ex Vivo with Electrical Impedance Spectroscopy

**DOI:** 10.2478/joeb-2021-0011

**Published:** 2021-11-29

**Authors:** Jie Hou, Runar Strand-Amundsen, Stina Hødnebø, Tor Inge Tønnessen, Jan Olav Høgetveit

**Affiliations:** 1 Department of Clinical and Biomedical Engineering, Oslo University Hospital, 0424 Oslo, Norway.; 2 Department of Physics, University of Oslo, 0316 Oslo, Norway.; 3 Department of Emergencies and Critical Care, Oslo University Hospital, 0424 Oslo, Norway.; 4 Institute of Clinical Medicine, University of Oslo, 0318 Oslo, Norway.

**Keywords:** Bioimpedance, Human small intestine, Cole model, *P_y_* value

## Abstract

Electrical impedance spectroscopy is a well-established tool for monitoring changes in the electrical properties of tissue. Most tissue and organ types have been investigated in various studies. As for the small intestine, there are several published studies conducted on pig and rat models. This study investigates the changes in passive electrical properties of the complete wall of the human intestine non-invasively during ischemia. We aim to use the passive electrical properties to assess intestinal viability. The bioimpedance measurements were performed using a two-electrode set-up with a Solartron 1260 Impedance/gain-phase analyser. The small intestinal samples were resected from patients who underwent pancreaticoduodenectomy. Impedance measurements were conducted following resection by placing the electrodes on the surface of the intestine. A voltage was applied across the intestinal sample and the measured electrical impedance was obtained in the ZPlot software. Impedance data were further fitted into a Cole model to obtain the Cole parameters. The *P_y_* value was calculated from the extracted Cole parameters and used to assess the cell membrane integrity, thus evaluate the intestinal viability. Eight small intestinal segments from different patients were used in this study and impedance measurements were performed once an hour for a ten-hour period. One hour after resection, the impedance decreased, then increased the next two hours, before decreasing until the end of the experiment. For all the intestinal segments, the *P_y_* values first increased and reached a plateau which lasted for 1 - 2 hours, before it decreased irreversibly. The time interval where *P_y_* value reached the maximum is consistent with reported viable/non-viable limits from histological analysis.

## Introduction

Assessment of intestinal viability following acute intestinal ischemia is important. There is an associated high mortality rate (30 - 93 %) partly due to the difficulty in diagnosing and treating the disease before irreversible injury occurs [1, 2, 3]. Following intestinal ischemia, estimation of ischemia time and determination of resection region(s) play a decisive role. The main challenge in detecting intestinal ischemia is the diffuse symptoms. It is often hard to determine how long the intestine has been ischemic and if it is still viable or not. Visual inspection and palpation are still the standard clinical methods for evaluation of the intestinal viability. Estimation of color change, presence of visible peristalsis and bleeding from cut edges are often used to estimate the intestine condition [4, 5, 6, 7, 8]. Those methods are non-specific and often unreliable. Viable tissue might be removed, or more critically, irreversibly damaged tissue might be left in the patient. This may lead to a second surgery and slow down the patient recovery. Moreover, histological analysis has been used to assess intestinal viability [9] and a number of studies have been published. They report that the time before irreversible injury occurs varies between species, anatomical locations and between the ischemia models used [10, 11, 12]. There is no standard classification method for the histological assessment of intestinal ischemia injury [13]. Therefore, a non-invasive, easy to employ and reliable monitoring method of ischemic tissue injury is of great interest [14].

Electrical impedance spectroscopy (EIS) has been regarded as a promising method for early diagnosis and monitoring of tissue ischemia due to its non-invasive, non-destructive and easily applicable nature [14, 15].

Over the past decades, EIS has been utilized to investigate changes in electrical parameters during ischemia in various tissues [16, 17, 18, 19, 20]. The frequency dependent EIS measures how well materials impede electric current flow, when a voltage is being applied to the material under test. Biological materials contain cells with intra-and extra-cellular fluid. At low frequencies the current flow is mainly in the extracellular fluid, while with increasing frequency, the current passes through the cell membranes, resulting in different electrical properties [21]. The impedance response of biological tissue is strongly influenced by the cell composition [22]. During ischemia, physical changes in the tissue results in altered impedance values. As ischemia prolongs, there is a shift in the ratio between extracellular and intracellular water. The amount of extracellular water decreases, when the cells swell due to osmosis as the ionic pump function declines [21, 23]. Thereafter, a decrease of impedance is observed due to increased cell membrane permeability caused by reduced functionality [24]. As ischemia time prolongs, the *β*-dispersion disappears over time as the cell membranes collapse [21].

In this work, we investigated how electrical parameters of the human small intestine changed during ischemia and the data were fitted to a Cole model and analyzed. Using the Cole equation, the measured impedance data can both be visualized and analysed through the Cole parameters [25]. The *P_y_* value obtained from the Cole parameters can be used to evaluate the cell membrane integrity [26] to assess the ischemic injury of the small intestine.

## Materials and methods

A total of 8 experiments were conducted on segments of resected human small intestine. The segments came from volunteer participants undergoing standard pancreaticoduodenectomy (Whipple procedure). The study involved no extra surgical procedures for the patients. The surgeon removed the head of the pancreas and a part of the small intestine. We received the intestine segments (length range 10 - 15 cm) immediately after the resection.

### Experimental set-up

Impedance measurements were performed with a Solartron 1260 Impedance/gain-phase analyser (Solartron Analytical, UK) with a 1294A interface. A pair of Ag/AgCl electrocardiography disc electrodes with 9 mm in diameter (Quickels System AB, Sweden) was connected to the gain-phase analyzer. During the measurements, the data were logged by the ZPlot software (Scribner Associates, USA). The two-electrode set-up was chosen based on the previous study conducted on porcine small intestine by Strand-Amundsen et al. [27]. The reason for not choosing a tetrapolar set-up is that the set-up is more vulnerable to errors compared to bipolar set-ups [21]. Using a tetrapolar set-up on the surface of the small intestine, the current can easily shunt across the surface of the pick-up electrodes. This brings the potential of the pick-up electrodes to a level that is closer to that of the current-carrying electrodes. This leads to an overestimation of the transfer impedance and can lead to measuring a positive phase. A 50 mV signal was applied during all the measurements, while the resulting current was measured and calculated into impedance values. The frequency range used was from 100 Hz to 1 MHz, with 41 log-spaced frequency points. The resected intestinal samples were kept inside a container with a Ringer-solution with 4 % Albumin. The container was placed in a temperature controlled water bath at a constant temperature of 37 °C (normal bowel temperature) between the measurements in order to maintain a stable temperature and reduce loss of humidity.

**Figure 1:**
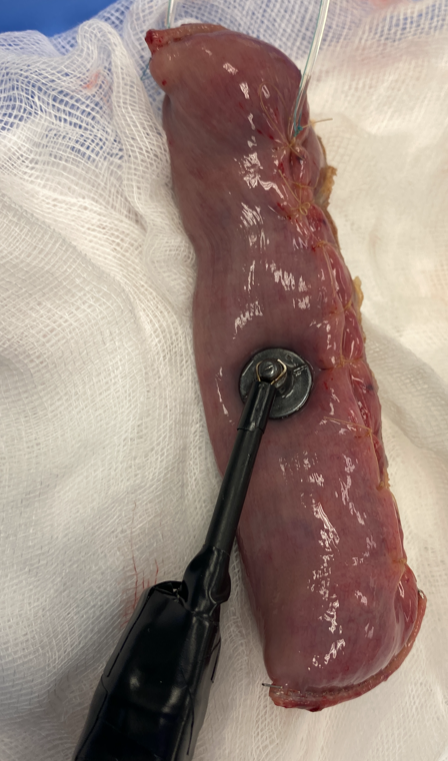
Experiment set-up. Human intestine sample under measurement.

### Ethical approval

The research related to human use has been complied with all relevant national regulations, institutional policies and in accordance with the tenets of the Helsinki Declaration, and has been approved by the Norwegian regional ethical committee (2018/1630).

### Data analysis

Electrical impedance is the ability of a material to oppose current flow. Complex electrical impedance (*Z*) is expressed as follows:


(1) Z=R+jX

The real part of the impedance is the in-phase resistance (*R*) which in tissue is mainly due to free ions, whereas the imaginary part is the capacitive reactance (*X*), mainly resulting from capacitive effects of the cell membranes. The impedance modulus *∣Z∣* can be expressed as:


(2) $$|Z|=\sqrt{R^{2}+X^{2}}$$

In 1940, Cole [28] introduced a mathematical model that can be used to fit the experimentally obtained electrical impedance data (Equation (3)). The model is based on four parameters *R*_0_, *R_1_*, *α* and *τ*:


(3) $$Z(\omega )=R_{\infty }+\frac{R_{0}-R_{\infty }}{1+(j\omega \tau {)}^{\alpha }}$$

The Cole equation can be separated into its real (resistance (*R*)) and imaginary (reactance (*X*)) component as a function of the angular frequency *ω*.


(4) $$R(\omega )=R_{\infty }+\frac{\left(R_{0}-R_{\infty }\right)\left(1+(\omega \tau {)}^{\alpha }\cos\left(\frac{\alpha \pi }{2}\right)\right)}{1+2(\omega \tau {)}^{\alpha }\cos\left(\frac{\alpha \pi }{2}\right)+(\omega \tau {)}^{2\alpha }}$$

where *R*_0_ and *R_1_* are resistance at very low and very high frequencies, respectively. *α* is a dimensionless shape parameter which takes value between 0 and 1. *τ* is the time constant. *ω* is the angular frequency (*ω* = 2*πf* ).

Often, it is not necessary to use the entire frequency spectrum as there is a strong correlation between the impedance values at adjacent frequencies [21]. Therefore, to assess the viability of the small intestine tissue, the *P_y_* value was chosen as it is a fast and direct measure of the *β*-dispersion (caused by cell membrane structures). It indicates the cell volume fraction in tissue and represents the cell membrane behavior [29]. As the cell membrane loses its integrity, the *β*-dispersion will gradually disappear resulting in a decreased *P_y_* value [29]. According to Pliquett et al. [29], the *P_y_* value can be calculated as the following:


(5) $$X(\omega )=-j\frac{\left(R_{0}-R_{\infty }\right)(\omega \tau {)}^{\alpha }\sin\left(\frac{\alpha \pi }{2}\right)}{1+2(\omega \tau {)}^{\alpha }\cos\left(\frac{\alpha \pi }{2}\right)+(\omega \tau {)}^{2\alpha }}$$

(6) $$P_{y}=\frac{R_{0}-R_{\infty }}{R_{0}}\times 100$$

*R*_0_ and *R_1_* can be estimated by fitting the measured data to the Cole equation (3). The *R*_0_ is related to the extracellular water since low frequency current is not able to pass through the cell membrane, whereas the *R_1_* can be used to estimate the intracellular water as high frequency current passes through the cell membranes [30]. Physically, the *P_y_* monotonically increase with the cell volume fraction surrounded by intact cell membranes [29]. When cells swell, the *P_y_* will typically increase as the cell volume fraction increases, whereas when cell membrane disintegrate, due to for instance cell death, the *P_y_* value will decrease as the volume fraction surrounded by insulating cell membranes decreases. The *P_y_* value is zero when no cells are present, and approaches one (100 %) when cells are intensely packed with no extracellular space [29].

## Results

### Impedance data analysis

**Figure 2:**
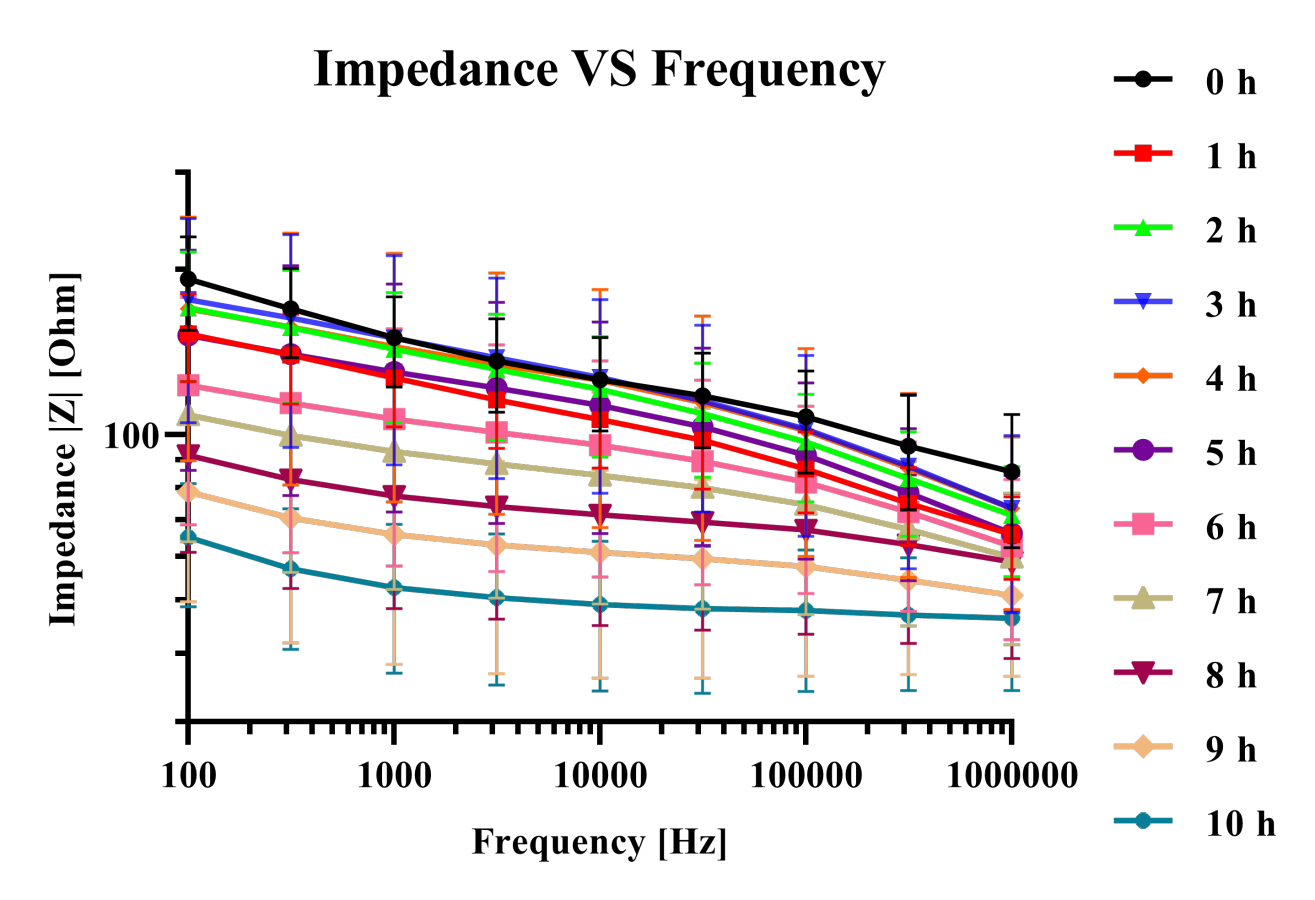
Plot of mean impedance values with 95 % confidence interval against frequency for 0 to 10 hours duration.

**Figure 3:**
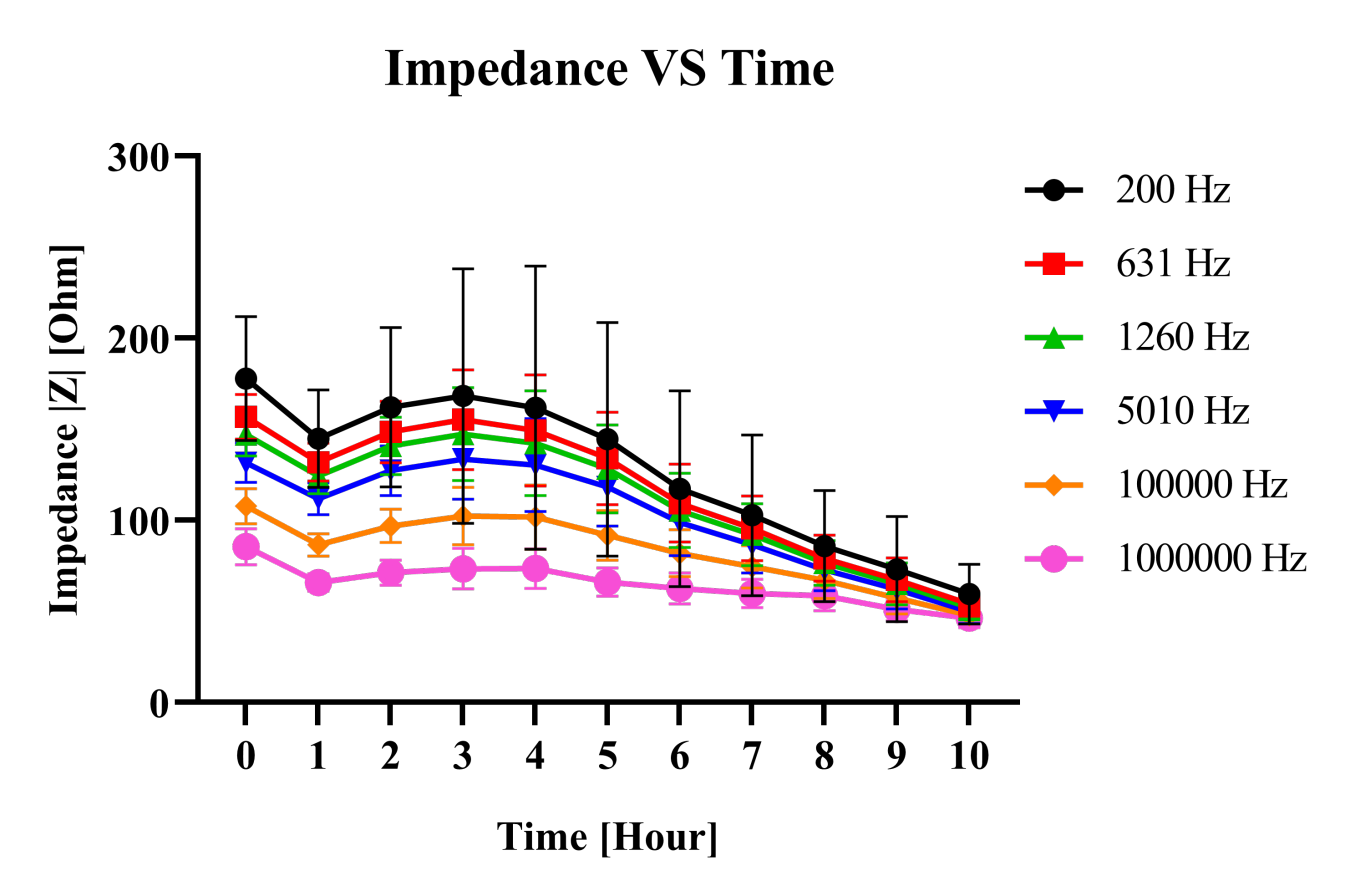
Plot of mean impedance values with 95 % confidence interval against time (0 to 10 hours) for selected frequencies.

The impedance of the small intestine was measured once an hour for a ten-hour period. The frequency dependent impedance showed different behavior for different degrees of ischemic injury. Figure 2 shows the mean measured impedance with 95 % confidence interval of eight samples (length range 10 - 15 cm) from eight different patients. The impedance values over the whole frequency range first decreased after one hour following resection, then increased for the two following hours, before decreasing again for the rest of the experiment. Comparing the mean impedance value from freshly resected samples with the mean impedance value after ten hours of ischemia, the decrease in impedance are 66.15 % (at the highest frequency) and 44.84 % (at the lowest frequency). Figure 3 shows the mean impedance values with 95 % confidence interval at selected frequencies. At all frequencies, the impedance value decreased after one hour. Then a plateau was observed at 3 hours before a continuous decrease until the end of the experiment. After 10 hours of ischemia, the impedance value all decreased towards 55 for all selected frequencies.

### Py value

**Figure 4:**
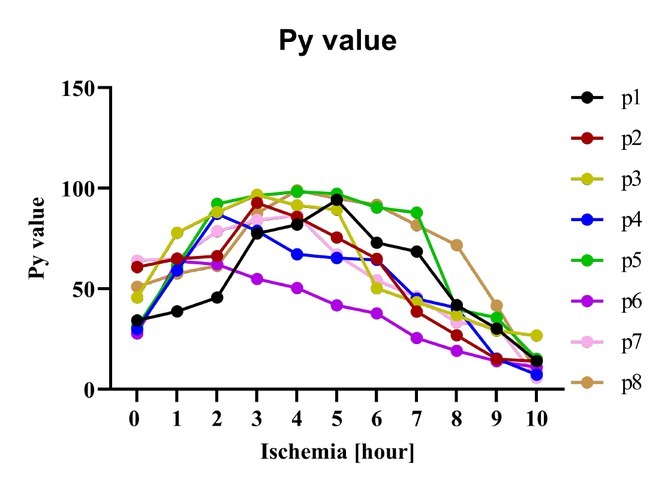
Plot of calculated *P_y_* value for eight small intestine samples from different patients.

The *P_y_* values were calculated from the Cole fitting parameters. Each impedance measurement was fitted separately, and we obtained one *P_y_* value for each sample and each time interval. Figure 4 shows the calculated *P_y_* values from the fitting procedure for eight intestine samples. The *P_y_* value first increased then decreased with time. For almost all samples, the *P_y_* value reached its maximum value between 2 - 4 hours.

## Discussion

The determination of the viability of ischemic intestine is still performed by manual observation of non-specific parameters like color, peristalsis and bleeding. We investigated how the *P_y_* value obtained from electrical impedance measurements associate with tissue vitality based on histological evaluation in a prior study [31]. As the *P_y_* value is a direct measure of the contributions of the cell membranes, it can be used to directly assess the ischemic injury levels in tissue. Ischemic injury is well known to directly affect cell membranes through several mechanisms, influencing the passive electrical properties of the cells. Therefore, ischemic injury can be sensitively detected by passive electrical properties [26].

As figure 4 shows, the *P_y_* value first increased. This associates with the cell swelling due to reduced function in the ionic pumps during the early phase of ischemia. During ischemia there is also an accumulation of metabolites, which also influence cell swelling. The cell swelling leads to a ratio change between intracelluar and extracelluar liquids [21, 32]. Thereafter, the *P_y_* value decreased with time as cell membrane loses its integrity. The time maximum *P_y_* value occurs corresponds to the reported viable and non-viable state transition of the tissue. Pliquett et al. [26] proposed that *P_y_* value increases by ischemic stress and plateaus for a period of time. Thereafter, it decreases continuously due to dissociation of the cell membranes and destruction of tissue. Strand-Amundsen et al. [31] reported, based on histological analysis, that irreversible injury on pig jejunum occurs around 3 - 4 hours of ischemia following reperfusion. However, histological assessment is difficult, due to the heterogeneous nature of the intestine sample [33, 34].

Interestingly, for almost all samples, the *P_y_* value reached the maximum value between 2 - 4 hours, which is consistent with the reported histological analysis results. Pliquett et al. [26] suggested that the *P_y_* value is good for assessment of tissue vitality, where the tissue is vital until the maximum is reached, followed by an irreversible decrease.

Another study published by Chen et al. [35] concluded that ileum segments can tolerate ischemia for less than 2 hours. Beltran et al. [36] reported that intestinal segments subjected to reperfusion after 2 hours of ischemia, did not fully recover after superior mesenteric artery occlusion in pig models. Regarding the ischemia tolerance time, based on the limited references, it appears that our *P_y_* value corresponds well with the estimated small-intestinal viability limits reported in the above mentioned studies.

Our results show that the *P_y_* value varies between individuals. This can be due to several factors including age, weight, body mass index (BMI) and medical history. Especially, a high BMI value may affect the impedance measurements as fat tissue has different electrical properties. In addition, some of the intestinal samples were from patients that previously had received chemotherapy. Chemotherapy can influence the measured impedance, for instance, due to fibrosis in parts of the tissue. As intestinal injury caused by ischemia appears to be heterogeneous [27], the measurement site where the electrodes are placed on the tissue may not represent the ischemic injury in the whole intestinal sample. Further on, there might be influences from the variation of mechanical pressure between the two electrodes during the measurements. When exposed to ischemia for more than approximately 3 hours, the intestinal wall gradually appears thinner, as the muscle cells are unable to function. Thus, the intestinal segment becomes softer, resulting in the surface of the intestine being more flexible to the mechanical pressure from the electrodes. This leads to a narrower distance between the electrodes, possibly changing the current path through the intestinal tissue. Another factor that can cause difference between the measurements is temperature variation. To prevent temperature to influence the measurements, the intestinal segment was kept in a container with constant temperature between the measurements. The temperature changes from exposing the intestinal segment to the environment for one or two minutes during measurements can be considered negligible.

To the best of our knowledge, this is the first study to investigate ischemic injury in human small intestine non-invasively using EIS and *P_y_* value. Strand-Amundsen et al. [37] performed bioimpedance measurements on pig small intestine ex vivo, where they found that there was an initial increase in impedance during the first hour, followed by a stable period of two hours, before the impedance started to decrease. The electrical behavior observed by [37] was different for the first two hours compared with our observations. We found that the impedance first decreased after one hour of ischemia, then increased for the following two hours. The reason behind these differences could be related to the temperature difference between the first and second measurements that we performed, where the temperature was lower during the first measurement. The intestinal sample was exposed to air for several minutes after the resection before we performed our first measurement. For the following measurements the temperature was kept constant in a water bath. Another possible cause of the observations could be due to potential variation in the development in passive electrical properties during ischemia between pig and human.

Evaluation of changes in the passive electrical properties of human intestine during ischemia in eight intestinal segment is a limitation. A small dataset may not reveal the true variation in the group. We plan to increase the number of intestine samples in the future. As for the measurement techniques, placing the electrodes against the intestine wall results in variations of pressure which can potentially affect the measured impedance. Efforts were made to control this variation, but it still cannot be completely excluded. There are also some uncertainties regarding the reproducibility of the impedance data. The reported reproducibility of passive electrical behavior within the same tissue in the same animal model is not better than 90–95 % [29].

## Conclusion

From our limited dataset, it is apparent that electrical properties can be used to assess the degree of ischemic injury on the human intestine. Our results suggest that there is a difference in the early changes during ischemia in measured impedance between pig intestine and human intestine. The time development of the *P_y_* parameter, which assesses the integrity of cell membranes, associated with the time development of ischemic injury reported in other studies. An increasing *P_y_* value associated with reported cell swelling, while a decreasing *P_y_* value associated with reported increase in leakage from- and dissociation of- the cell membranes in the later ischemia process. Conclusively, the *P_y_* value can, comparably to histological analysis, be used to evaluate the viability of the intestine, where the tissue is vital until the maximum *P_y_* value is reached.

## References

[1] Bala M, Kashuk J, Moore EE, Kluger Y, Biffl W, Gomes CA, Ben-Ishay O, Rubinstein C, Balogh ZJ, Civil I, Coccolini F, Leppaniemi A, Peitzman A, Ansaloni L, Sugrue M, Sartelli M, Di Saverio S, Fraga GP, Catena F (2017). Acute mesenteric ischemia: guidelines of the World Society of Emergency Surgery. World Journal of Emergency Surgery.

[2] Brandt LJ, Boley SJ (2000). AGA technical review on intestinal ischemia. en. Gastroenterology.

[3] Tilsed JVT, Casamassima A, Kurihara H, Mariani D, Martinez I, Pereira J, Ponchietti L, Shamiyeh A, al-Ayoubi F, Barco LAB, Ceolin M, D’Almeida AJG, Hilario S, Olavarria AL, Ozmen MM, Pinheiro LF, Poeze M, Triantos G, Fuentes FT, Sierra SU, Soreide K, Yanar H (2016). ESTES guidelines: acute mesenteric ischaemia. en. European Journal of Trauma and Emergency Surgery.

[4] Bryski MG, Frenzel Sulyok LG, Kaplan L, Singhal S, Keating JJ (2020). Techniques for intraoperative evaluation of bowel viability in mesenteric ischemia: A review. en. The American Journal of Surgery.

[5] Reginelli A, Iacobellis F, Berritto D, Gagliardi G, Di Grezia G, Rossi M, Fonio P, Grassi R (2013). Mesenteric ischemia: the importance of differential diagnosis for the surgeon. BMC Surgery.

[6] Herbert GS, Steele SR (2007). Acute and Chronic Mesenteric Ischemia. en. Surgical Clinics of North America. Vascular Surgery.

[7] Orland PJ, Cazi GA, Semmlow JL, Reddell MT, Brolin RE (1993). Determination of Small Bowel Viability Using Quantitative Myoelectric and Color Analysis. en. Journal of Surgical Research.

[8] Bulkley GB, Zuidema GD, Hamilton SR, O’mara CS, Klacsmann PG, Horn SD (1981). Intraoperative Determination of Small Intestinal Viability following Ischemic Injury: A Prospective, Controlled Trial of Two Adjuvant Methods (Doppler and Fluorescein) Compared with Standard Clinical Judgment. en-US. Annals of Surgery.

[9] Urbanavicius L, Pattyn P, Van de Putte D, Venskutonis D (2011). How to assess intestinal viability during surgery: A review of techniques. World Journal of Gastrointestinal Surgery.

[10] Brolin RE, Bibbo C, Petschenik A, Reddell MT, Semmlow JL (1997). Comparison of ischemic and reperfusion injury in canine bowel viability assessment. en. Journal of Gastrointestinal Surgery.

[11] Haglund U, Bulkley GB, Granger DN. (1987). On the pathophysiology of intestinal ischemic injury. Clinical review. eng. Acta Chirurgica Scandinavica.

[12] Gonzalez LM, Moeser AJ, Blikslager AT Animal models of ischemia-reperfusion-induced intestinal injury: progress and promise for translational research. American Journal of Physiology-Gastrointestinal and Liver Physiology 2015 Jan; 308.

[13] Quaedackers JS, Beuk RJ, Bennet L, Charlton A, Egbrink MG oude, Gunn AJ, Heineman E (2000). An evaluation of methods for grading histologic injury following ischemia/reperfusion of the small bowel. eng. Transplantation Proceedings.

[14] Mellert F, Winkler K, Schneider C, Dudykevych T, Welz A, Osypka M, Gersing E, Preusse CJ Detection of (Reversible) Myocardial Ischemic Injury by Means of Electrical Bioimpedance. IEEE Transactions on Biomedical Engineering 2011Jun; 58.

[15] Yang Y, Ni W, Sun Q, Wen H, Teng Z (2013). Improved Cole parameter extraction based on the least absolute deviation method. eng. Physiological Measurement.

[16] Martinsen Ø, Grimnes S, Mirtaheri P (2000). Non-invasive measurements of post-mortem changes in dielectric properties of haddock muscle – a pilot study. Journal of Food Engineering.

[17] Gheorghiu M, Eberhard G (2002). Revealing alteration of membrane structures during ischema using impedance spectroscopy. Songklanakarin Journal of Science and Technology.

[18] Salazar Y, Cinca J, Rosell-Ferrer J (2004). Effect of electrode locations and respiration in the characterization of myocardial tissue using a transcatheter impedance method. eng. Physiological Measurement.

[19] Ivorra Cano A (2005). Contributions to the measurement of electrical impedance for living tissue ischemia injury monitoring. eng. Accepted: 2011-04-12T15:12:40Z ISBN: 9788468913544 Publication Title: TDX (Tesis Doctorals en Xarxa). Ph.D. Thesis.

[20] Asami K (2007). Dielectric properties of biological tissues in which cells are connected by communicating junctions. English. Journal of Physics. D, Applied Physics.

[21] Grimnes S, Martinsen ØG Bioimpedance and Bioelectricity Basics. Academic Press 3rd Edition.

[22] Mouritsen OG, Bloom M (1993). Models of lipid-protein interactions in membranes. eng. Annual Review of Biophysics and Biomolecular Structure.

[23] Strand-Amundsen RJ, Tronstad C, Kalvøy H, Gundersen Y, Krohn CD, Aasen AO, Holhjem L, Reims HM, Martinsen ØG, Høgetveit JO, Ruud TE, Tønnessen TI. (2016). In vivo characterization of ischemic small intestine using bioimpedance measurements. eng. Physiological Measurement.

[24] Schäfer M, Schlegel C, Kirlum HJ, Gersing E, Gebhard MM (1998). Monitoring of damage to skeletal muscle tissues caused by ischemia. en. Bioelectrochemistry and Bioenergetics.

[25] Ayllon D, Seoane F, Gil-Pita R (2009). Cole equation and parameter estimation from electrical bioimpedance spectroscopy measurements - A comparative study. eng. Conference proceedings: ... Annual International Conference of the IEEE Engineering in Medicine and Biology Society. IEEE Engineering in Medicine and Biology Society. Annual Conference.

[26] Pliquett F, Pliquett U (1999). Stress Action on Biological Tissue and Tissue Models Detected by the Py Value. en. Annals of the New York Academy of Sciences.

[27] Strand-Amundsen RJ, Tronstad C, Kalvøy H, Gundersen Y, Krohn CD, Aasen AO, Holhjem L, Reims HM, Martinsen ØG, Høgetveit JO, Ruud TE, Tønnessen TI. (2016). In vivocharacterization of ischemic small intestine using bioimpedance measurements. en. Physiological Measurement.

[28] Cole KS, Cole RH Dispersion and Absorption in Dielectrics I. Alternating Current Characteristics. The Journal of Chemical Physics 1941 Apr; 9.

[29] Pliquett U, Altmann M, Pliquett F, Schöberlein L (2003). Py—a parameter for meat quality. en. Meat Science.

[30] Alves J, Sousa F, Correia MV (2018). Calibration and Electrical Validation of a BIS Portable System. eng. Studies in Health Technology and Informatics.

[31] Strand-Amundsen RJ, Reims HM, Reinholt FP, Ruud TE, Yang R, Høgetveit JO, Tønnessen TI (2018). Ischemia/reperfusion injury in porcine intestine - Viability assessment. World Journal of Gastroenterology.

[32] Sun J, Zhang R, Zhang Y, Liang Q, Zhang F, Xu P, Li G (2020). Evaluation of fish freshness using impedance spectroscopy based on the characteristic parameter of orthogonal direction difference. en. Journal of the Science of Food and Agriculture.

[33] Hillman H (2000). Limitations of clinical and biological histology. en. Medical Hypotheses.

[34] Dabareiner RM, Sullins KE, White NA, Snyder JR (2001). Serosal Injury in the Equine Jejunum and Ascending Colon After Ischemia-Reperfusion or Intraluminal Distention and Decompression. en. Veterinary Surgery.

[35] Chen SH, Tang YB, Chen HC (2013). Survival of Transferred Ileum after Ischemia Time Longer than 1 Hour: A Clinical Result Different from Animal Studies. en. Journal of the American College of Surgeons.

[36] Beltran NE, Sacristan E Gastrointestinal ischemia monitoring through impedance spectroscopy as a tool for the management of the critically ill. en. Experimental Biology and Medicine 2015 Jul; 240.

[37] Strand-Amundsen RJ, Reims HM, Tronstad C, Kalvøy H, Martinsen ØG, Høgetveit JO, Ruud TE, Tønnessen TI (2017). Ischemic small intestine—in vivoversusex vivobioimpedance measurements. en. Physiological Measurement.

